# The Effect of Fermentation of High- or Low-Tannin Fava Bean on Glucose Tolerance, Body Weight, Cardiovascular Function, and Blood Parameters in Dogs After 7 Days of Feeding: Comparison With Commercial Diets With Normal vs. High Protein

**DOI:** 10.3389/fvets.2021.653771

**Published:** 2021-05-11

**Authors:** Luciana G. Reis, Tressa Morris, Chloe Quilliam, Lucas A. Rodrigues, Mathew E. Loewen, Lynn P. Weber

**Affiliations:** ^1^Department of Veterinary Biomedical Sciences, Western College of Veterinary Medicine, University of Saskatchewan, Saskatoon, SK, Canada; ^2^Department of Animal and Poultry Science, College of Agriculture and Bioresources, University of Saskatchewan, Saskatoon, SK, Canada; ^3^Prairie Swine Centre, Inc., Saskatoon, SK, Canada

**Keywords:** fava bean (*Vicia faba*), *Candida utilis*, fermentation, cardiovascular function, blood chemistry, glucose tolerance, domestic dog (*Canis familiaris*)

## Abstract

Fava bean, which is available in high- and low-tannin varieties, is not an approved pet food ingredient and was not included in the “assumed to be safe” category based on its ability to cause favism and hemolytic anemia in susceptible humans. The effects of 7-day feeding of test canine diets containing moderate protein (~27%) were compared with two control commercial diets with normal (NP, grain-containing, ~25% protein) or high protein (HP, grain-free, ~41% protein). Fava bean diets were formulated either with or without *Candida utilis* fermentation processing to reduce antinutritional factors. Glucose tolerance, body weight, cardiovascular function, and blood parameters were investigated in beagles fed the NP or HP diets or a randomized, crossover, 2 × 2 Latin square design of the fava bean diets: unfermented high-tannin (UF-HT), fermented high-tannin (FM-HT), unfermented low-tannin (UF-LT), and fermented low-tannin (FM-LT). After 7 days, HP decreased red blood cells (RBC) (*P* < 0.05) compared with NP, while FM increased RBC compared with UF. HP increased blood bicarbonate, calcium, phosphorus, urea, cholesterol, and albumin:globulin ratio while decreasing bilirubin, liver enzymes, and total protein. Sodium:potassium ratio was increased in UF-HT, decreased in FM-HT, and intermediate in LT regardless of fermentation. Blood phosphorus was increased in HT. Blood amylase was increased in FM-HT and decreased in FM-LT, being intermediate in UF regardless of fava bean variety. Blood direct bilirubin was decreased in HT regardless of fermentation. Of note, left ventricular end-systolic volume and cardiac output were increased in NP compared with HP-fed dogs, but were normal and had no significant differences among the fava bean diets. As expected, plasma taurine, cystine, and cysteine levels were increased in HP- compared with NP-fed dogs. Plasma cysteine levels were increased in HT- compared with LT-fed dogs and in FM- compared with UF-fed dogs. Taken together, these results show that fava bean appears to be safe as a dog food ingredient at least in the short term, and its nutritional value appears improved by fermentation. Moreover, blood chemistry parameters and cardiovascular function were impacted by protein content which merits further investigation with longer term feeding trials.

## Introduction

Fava bean (*Vicia faba* L.) has been regarded as a healthy, sustainable alternative for partially replacing animal protein sources in human diets (1). The varieties of fava bean are divided by their tannin levels such as low or normal/high tannin content which affects taste (2). Because pet owners are increasingly matching their own nutritional choices with that of their pets, incorporation of fava beans in pet diets as a source of carbohydrates and protein has been considered. Fava bean safety in dogs has been scarcely explored and is not approved as a feed ingredient for pet food by the American Association of Feed Control Organization (AAFCO) yet due to concerns about potential toxicity from antinutritional factors (3). Further complicating the approval of a novel pulse ingredient such as fava beans for use in pet food, the US Food and Drug Administration (FDA) reported in July 2018, cases in which dilated cardiomyopathy (DCM) was observed in dogs fed grain-free diets, i.e., food formulated with potatoes and pulse ingredients instead of grains (4). DCM is recognized as the second most common type of genetically linked cardiac disease in the dog and is most prevalent in large or giant breeds (5). Dobermans, Boxers, Great Danes, Newfoundlands, Irish Wolfhounds, English Cocker Spaniels, and Portuguese Water Dogs are the breeds with the highest prevalence of DCM (6–9). Moreover, Golden Retrievers and American Cocker Spaniels have recently emerged as being predisposed to taurine deficiency (10, 11). DCM is described as a primary myocardial disorder causing systolic dysfunction with secondary ventricular dilation, regular or decreased wall thickness, and increased cardiac mass due to myocyte enlargement (12). However, while pulse ingredients in grain-free diets have been suggested as causally linked to DCM, the actual link between them has not been definitively demonstrated to date (13–15). Despite an acknowledgment by the FDA in November 2020 that the link between DCM and diet in dogs is multi-factorial and not due solely to pulses, this issue remains unresolved, leaving veterinarians, and pet owners wary of pulse-containing dog foods.

The best evidence so far for a link between DCM and nutrition in dogs is through taurine insufficiency (16, 17). Taurine is important to cardiac health because it participates in the reabsorption of calcium by the sarcoplasmic reticulum and enhances the sensitivity of the myofilaments to calcium (18). Therefore, as reviewed by Mansilla et al. (13), because calcium is a key component of cardiac contraction, in cases of taurine absence due to reduced synthesis or low intake of taurine and/or its precursors, the cardiac muscle tissue is unable to properly contract and presumably develops DCM. Dog diets do not require taurine and taurine is not considered essential since dogs can synthesize taurine from sulfur-containing amino acids such as cysteine and methionine (19). Grain-free and pulse-containing diets are higher in fermentable fiber which has been demonstrated to decrease protein digestibility though either an increased fermentation of the sulfur-containing amino acids leading to decreased bioavailability or to higher fecal excretion (20, 21). Previous studies have reported that high fermentable dietary fiber in dogs increases the need for dietary taurine or taurine precursors (e.g., methionine and cysteine) due to increased fecal excretion of taurocholate (the predominant bile acid in dogs), leading to fecal loss of taurine (22, 23).

Fava beans have numerous antinutritional factors such as condensed tannins, trypsin inhibitor activity, lectins, and pyrimidine glucosides (24). Tannins are known to cause reduction of protein and energy digestibility (3). The pyrimidine glucosides (vicine and convicine) lead to favism which is a blood disorder caused when fava beans are eaten by humans with genetic mutations of glucose-6-phosphate dehydrogenase (G6PD), leading to decreased G6PD activity and reduced ability of red blood cells (RBCs) to produce ATP and regenerate glutathione (25). This then makes the red blood cell susceptible to oxidative damage, leading to rapid RBC death and acute anemia when uncooked fava beans are consumed. There are no reports of favism in dogs to the best of our knowledge. However, because of this concern, fava beans cannot be placed in the “generally assumed to be safe” category by AAFCO and thus is not currently used in pet food until proven otherwise.

Fermentation is a processing technique well-known for improving health, functional, and nutraceutical effects of foods (26). Fermentation also has been positively associated with enhanced nutritional quality of pulses by reducing their levels of anti-nutritional factors (27–29). For example, fermentation of fava bean with *Lactobacillus plantarum* reduced the content of the antinutritional factors vicine and convicine by more than 90% while increasing the amount of free amino acids and enhancing protein digestibility (30). Likewise, Rizzello et al. (31) demonstrated a complete degradation of the pyrimidine glycosides in *L. plantarum*–fermented fava bean flour after 48 h, which indicates the usefulness of bioprocessing techniques for industrial fava bean detoxification. In comparison, some yeast organisms in the *Candida* clade have the potential to synthesize and increase taurine content while other yeasts and bacteria do not (32). This current study explored the use of fermentation with the yeast *Candida utilis* to both reduce antinutritional factors and increase taurine content.

The objective of this study was to determine if short-term (7-day) feeding of beagles with a moderate protein diet that has 30% inclusion of fava bean flour would show altered glucose tolerance, body weight, cardiovascular function, and blood parameters when contrasted to commercial diets with normal vs. high protein. Two commercial diets with normal or high protein content and four different fava bean-containing diets with low- (LT) or high-tannin (HT) content were fed, both varieties with and without fermentation. We hypothesized that pulse-based diets would impair cardiovascular health due to the low taurine, cysteine, or methionine levels and high fiber content. Moreover, fermentation of fava bean flour with *C. utilis* would enhance diet quality and, consequently, health of dogs.

## Materials and Methods

### Fava Bean Ingredients and Fermentation Protocol

Low-tannin (Snowdrop) and HT (Florent) fava bean varieties, genotypes grown in Saskatchewan (2), were dehulled and ground into flour using a 400-μm screen. Fermentation of each variety was adapted from methodology used previously in pea flour in our laboratory (33). Briefly, *C. utilis* (ATCC 9950) was maintained in sterile 80% (v/v) glycerol solution at −80°C, then reactivated on YGC agar plates when needed (Yeast Extract Glucose Chloramphenicol Agar; catalogue number 95765; Sigma Aldrich, St Louis, MO). Seeded plates were incubated at 30°C for 72 h. Two loops of colonies were transferred using a flame-sterilized platinum needle to a 250-ml sterile conical flask containing 100 ml of YPD liquid medium (Yeast Peptone Dextrose—A1374501; ThermoFisher, Waltham, MA). The flask was incubated in a horizontal shaker incubator (30°C) at 120 rpm for 12 to 15 h. After this period, 10 ml of the cultured yeast mass was transferred into a 500-ml sterile conical flask containing 250 ml of YPD liquid medium. The medium containing the yeast was then incubated on a horizontal shaker (30°C) at 120 rpm for an additional 12 to 15 h. Twenty-kilogram batches of each fava bean variety were mixed with the yeast broth, ammonia, and sterile water to form a soft dough, then fermented in an adapted cement mixer with temperature maintained at 30°C. The fermentation slurry was mixed for 3 min every hour for 72 h. Samples were collected every 24 h, and serial dilutions of mixture plated on sterile agar plates, then incubated at 30°C for 72 h to verify yeast viability and count throughout the process. The fermented fava bean flour was subsequently dried in an oven (60°C) for 48 h at the WCVM before transport to the University of Saskatchewan Canadian Feed Research Centre (North Battleford, Canada) for grinding of the fermented flours, followed by mixing of all test diets (both fermented and unfermented fava bean flours), extruding, and vacuum coating with fat to produce the final dry kibble format of the diets to be used in the feeding trials.

### Animals and Diets

All animal use and procedures were approved by the University of Saskatchewan Animal Care Committee (Animal Utilization Protocol #20190055) and adhered to the Canadian Council on Animal Care. Eight neutered beagles, four males and four females, at ideal body weight (8.8 ± 1.9 kg) and a mean age of 2.6 ± 1.0 years, were obtained from King Fisher International (Toronto, ON, Canada) or Marshall Bioresources (North York, NY, USA) and housed at the Western College of Veterinary Medicine (Saskatoon, SK, Canada). The animals were housed individually in 1.1 × 2.7-m kennels with outdoor kennel access at night and during feeding but kept in a group kennel area during the day. Dogs were walked or socialized with volunteers for at least 1 h every day. When not on trial, dogs were fed a commercial adult maintenance dry diet (Purina Dog Chow; Ralston Purina Co, St Louis, MI) in amounts that maintained each individual dog at ideal condition (score of 4–6 on a 9-point scale) in conjunction with energy requirements stated in the National Research Council guidelines (34). All dogs were acclimated to procedures with rewards before the start of experiments to minimize stress, thus no anesthetics or sedatives were used in this study.

In total, six diets were tested. Low- or high-tannin fava bean flours were used at 30% inclusion, in either fermented or unfermented formats with diet formulations indicated in Supplementary Table 1. The four fava bean test diets were unfermented low-tannin [UF-LT; 27% crude protein (CP)], fermented low-tannin (FM-LT; 28% CP), unfermented high-tannin (UF-HT; 27% CP), and fermented high-tannin (FM-HT; 28% CP). Insoluble ash (Celite) was included at 1% as a non-digestible marker. Diets were formulated in accordance with the nutrient guidelines for adult dog maintenance set by AAFCO to be nutritionally balanced before being extruded under identical process conditions (35). The two additional diets tested were commercial diets containing normal-protein (NP; Purina Dog Chow, St. Louis, MI; 24% CP) or high-protein content (HP; GO! Solutions Carnivore, Richmond, BC; 41% CP). Ingredient lists for the commercial diets are shown in Supplementary Table 2. All six diets were randomly sub-sampled and sent for proximate analysis (Central Testing, Winnipeg, MB, Canada) (Supplementary Table 3). The analyzed content of crude fiber, non-fiber carbohydrates, metabolizable energy, vicine, and convicine of UF-LT, UF-HT, FM-LT, and FM-HT diets are 1.1, 0.5, 0.5, and 0.5%; 60.7, 62.1, 61.6, and 61.8%; 3.7, 3.8, 3.8, and 3.8 kcal/g; 1.8, 1.8, 0.4, and 0.6 mg/g; and 0.5, 0.6, 0.1, and 0.2 mg/g, respectively. The analyzed content of crude fiber, non-fiber carbohydrates, and metabolizable energy of NP and HP diets are 0.8 and 1.1%, 53.0 and 29.8%, and 4.0 and 4.1 kcal/g, respectively. Dogs were fed each diet for 7 days, with the NP or HP diets fed during the first and sixth weeks, respectively. From weeks 2 to 5, using a randomized, crossover, 2 × 2 Latin square design, the UF-LT, FM-LT, UF-HT, and FM-HT diets were fed. Commercial diets were not included in the crossover design because the fava bean–based diets were formulated to contain the same nutrient profile to enable a reasonable comparison. In contrast, NP and HP diets have different ingredients at unknown inclusion levels (specific formulation not available on label), making direct comparisons difficult. Dogs were weighed at the beginning of the experiment and after each feeding week. The amount of diet allotted per dog per day was calculated to be isocaloric with the daily requirement for that dog using standard equations that determine the energy requirements for individual dog maintenance [maintenance energy (ME in kcal)] = [(70 × BW^0.7^) × 1.6], daily portions divided, and dogs fed twice daily (at 08:30 and 16:30 h). Bowls were removed before the next meal and any uneaten food was weighed and recorded. All dogs generally consumed all food portioned in each meal within 5–10 min, with no palatability issues noted (Morris, Reis & Weber, unpublished).

### Digestibility Protocol

After a 5-day feeding period on each fava bean–based diet, feces were collected during the subsequent 2 days of each period (total of 7 days on each diet). Collected feces were labeled and frozen at −20°C until analysis. Feces were thawed, homogenized, and pooled by dog (i.e., two samples from each dog pooled per dietary treatment). Before laboratory testing, feces were dried in a forced air oven at 55°C for 72 h and ground in a cutting mill with a 1-mm sieve. Diets and feces were analyzed (Central Testing, Winnipeg, MB) according to AOAC standards (36) for dry matter by oven-drying the sample, non-fiber carbohydrates, crude protein applying the Kjeldahl method, and acid-hydrolyzed fat. Gross energy (GE) content of diets was determined using a bomb calorimeter. The equation below was used to calculate the apparent digestibility coefficients of dry matter, crude protein, non-fiber carbohydrates, fat, gross energy, methionine, cysteine, and taurine for each fava bean–based diet using Celite as a non-digestible marker (37):

$$\begin{array}{l} \text{Digestibility}\left(\text{\%}\right)=100-\left[100\times \left(\text{Mfeed}\times \text{Cfeces}/\text{Mfeces}\times \text{Cfeed}\right)\right] \end{array}$$

Where M feed and M feces represent concentrations of index compound in feed and feces, respectively; C feed and C feces represent concentrations of components of interest in feed and feces, respectively.

### Oral Glucose Tolerance Test and Blood Analysis

After 7 days of feeding each diet, dogs were fasted for 8 h then a syringe with 10 ml/kg body weight of a 10% glucose solution (1 g/kg BW glucose) fed to each dog by placing in the back of the mouth for an oral glucose tolerance test conducted at the same time each day. Before glucose feeding, the fasted dogs were aseptically catheterized using a peripheral intravenous catheter equipped with an extension tube inserted into the cephalic vein. Blood samples (~0.2 ml) were taken before feeding glucose (time 0) and at 15, 30, 60, and 90 min after feeding. Blood glucose was measured using a glucometer (OneTouch Ultra 2; LifeScan, Johnson & Johnson, New Brunswick, NJ) with a minimum of duplicate readings for each time or until two consistent readings were obtained. The extension tube was filled with blood and initial blood discarded before glucometer reading to ensure no contamination of blood with anticoagulant solution. Subsequently, after each blood sample was obtained, the catheter was flushed with a sterile citrate solution to prevent clotting. The trapezoidal method was used to determine the incremental area under the curve (AUC) for the glucose response (38). Peak concentration and time to peak concentration for glucose were also calculated.

Additional fasting blood samples (8 ml) were taken using collection tubes with and without EDTA. Complete blood cell count [red (RBC) and white (WBC) blood cell counts] and chemistry panel [cholesterol; total (TB), direct (DB), and indirect bilirubin (IB); alkaline phosphatase (ALP); alanine aminotransferase (ALT); creatine kinase (CK); gamma-glutamyltransferase (GGP); glutamate dehydrogenase (GLDH); total protein, albumin (A), globulin (G), and A:G] were analyzed at Prairie Diagnostic Services (Saskatoon, SK). Moreover, 3-ml subsamples of EDTA-tube blood from fasted animals were centrifuged at 2,000 rpm for 10 min for plasma collection. Plasma samples were kept at −80°C until further analyses of methionine, cystine, cysteine, and taurine content (UC Davis Amino Acid Lab, Davis, CA). Plasma amino acid concentrations were analyzed in an automated amino acid analyzer via cation-exchange high-pressure liquid chromatography separation and ninhydrin-reactive colorimetric detection (39–42).

### Cardiac Function, Blood Pressure, and Vascular Health

After 7 days of feeding each diet, dogs were tested for cardiovascular health. All ultrasound measurements were performed and analyzed by one individual. Before ultrasound, blood pressure was taken using a high-definition canine/feline oscillometer (VET HDO High Definition Oscillometer, Babenhausen, Germany). An average of two readings with good agreement was used to determine diastolic and systolic pressures. Endpoints of flow-mediated dilation were used as an indicator of vascular health and included brachial artery diameter during baseline, during inflation of a blood pressure cuff placed distal to the brachial artery, and at the time of peak dilation (30 s) after cuff release, as previously determined by our research group in dogs (43, 44). Echocardiography endpoints were used to assess cardiac function included heart rate (HR), stroke volume (SV), and cardiac output (CO) (44, 45). Moreover, left ventricular end-diastolic volume (EDV), left ventricular end-systolic volume (ESV), ejection fraction (EF), left ventricular diastolic free wall thickness (DWT), left ventricle systolic free wall thickness (SWT), systolic blood pressure (SBP), diastolic blood pressure (DBP), velocity time integral for bloodflow through the mitral valve (VTI), and maximum velocity of bloodflow through the mitral valve (MV) were also obtained. Flow-mediated dilation and echocardiography were measured using a SonoSite Edge II ultrasound (Fujifilm SonoSite, Bothell, WA) with detection using a P10x transducer (8–4 Hz) to detect cardiac endpoints and the L38xi (10–5 Hz) transducer for vascular imaging. Flow-mediated dilation was calculated using the following equation:

$$\begin{array}{r} \text{\%FMD}=100\text{\%}\times [(\text{maximum diameter postcuff release})\\ -\left(\text{baseline diameter}\right)]/\left(\text{baseline diameter}\right). \end{array}$$

Two-dimensional ultrasonography was used to measure left ventricular volume using the left parasternal apical two- and four-chamber views in diastole and systole (46). Two-dimensional guided M-mode echocardiography was used to obtain a right parasternal short-axis view of the heart at the level of the papillary muscles (46). Measurements of left ventricular end-diastolic inner diameter (LVID_d_) and left ventricular end-systolic inner diameter (LVID_s_) were also compiled from all dogs and normalized to body weight according to methodology described by Cornell et al. (47).

### Statistical Analysis

Analyses were performed using SAS (version 9.4; SAS Institute, Cary, NC). Before performing all analyses, the data were explored for normality and outliers using the PROC UNIVARIATE model in SAS and the Shapiro–Wilk test. One-way ANOVA was used to compare differences among diets among normal vs. high protein commercial diets and two-way ANOVA was used to compare parameters for fava bean–based diets (fixed effects being fava bean variety and fermentation). In the present study, we used mixed-sex dogs to control the variation possibly associated with this factor. Previous studies from our group have not detected sex-related differences among (spayed/neutered) dogs (44) and because sex-related differences are not related to the purposes of the present study, we did not include this factor in the model. All *post-hoc* analyses were performed using the Fisher least significant difference (LSD) method. Differences were considered significant at *P* < 0.05.

## Results

### Body Weight, Meal Portion, and Body Condition Score

Body weight (BW), BCS, and meal portion data are presented in Table 1. No significant effect (*P* > 0.05) of dietary protein content (NP vs. HP) was observed on BCS or BW after 7 days of feeding in beagles. Within commercial diets, meal portion (163–187 g/day) was significantly smaller (*P* < 0.05) in HP compared with NP-fed dogs to restrict meal size to an isocaloric amount among diets. Within fava bean–based diets, there was no effect of either FM, FB, or the interaction between FM and FB on body weight, BCS, or meal portion (*P* > 0.10).

**Table 1:** Body weight, food portion, and body condition score (BCS) of dogs fed diets formulated with either low or high tannin fava beans without (UF) or with (FM) fermentation, or normal (NP) vs. high (HP) protein commercial diets for 7 days each^*^.

**Item**	**Commercial**			**Low tannin**		**High tannin**			***P*****-value**^**a**^		
	**NP**	**HP**	**SEM**	**UF**	**FM**	**UF**	**FM**	**SEM**	***P***	**FB**	**FM**
Body weight (kg)	8.45	8.32	0.88	8.31	8.32	8.33	8.28	0.88	0.16	0.88	0.66
Food portion (g/day)	188	163	22.12	188	188	188	188	23.59	<0.01	1.00	1.00
BCS	4.62	4.50	0.18	4.56	4.62	4.55	4.50	0.18	0.35	0.34	0.95

* *Eight mixed-gender, neutered beagles were fed the NP or HP diets during the first and sixth weeks, respectively. From weeks 2 to 5, using a randomized, crossover, 2 × 2 Latin square design, 4 diets differing in fava bean variety and fermentation were compared as follows: unfermented (UF) high tannin, fermented (FM) high tannin, UF low tannin, and FM low tannin. Values expressed as means (n = 8). SEM = pooled standard error of the mean*.

a *P, P-value between commercial diets with different protein content (NP vs. HP); FB, fava bean (low tannin vs. high tannin); FM, fermentation (UF vs. FM). The interaction between FB and FM was not significant for any of the parameters measured (P > 0.10)*.

### Glucose Tolerance

**Figure 1:**
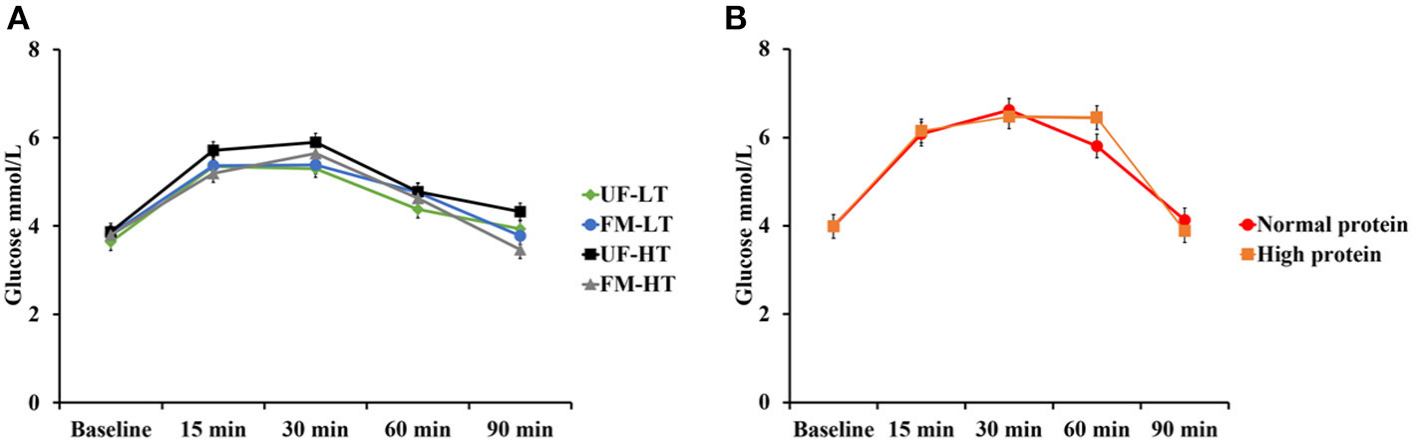
Blood glucose responses to the oral glucose tolerance test in dogs fed either low (LT) or high (HT) tannin fava bean–based diets without (UF) or with (FM) fermentation **(A)**, or normal vs. high protein commercial diets **(B)**. Eight mixed-gender, neutered beagles were fed the NP, or HP diets during the first and sixth weeks, respectively. From weeks 2 to 5, using a randomized, crossover, 2 × 2 Latin square design, 4 diets differing in fava bean variety and fermentation were compared as follows: UF-HT, FM-HT, UF-LT, and FM-LT. Fasting and peak glucose levels, time to peak, and area under the curve (AUC) were not influenced by dietary treatments (*P* > 0.10).

Time course of blood glucose responses to the oral glucose tolerance test are shown in Figure 1 with fasting and peak glucose levels (mmol/L), time to peak (min), and area under the curve (mmol/L × min) data shown in Table 2. There was no effect (*P* > 0.10) of dietary protein content (NP vs. HP) and no effect of FM, FB, or the interaction between FM and FB on fasting and peak glucose levels, time to peak, or AUC.

**Table 2:** Fasting and peak blood glucose levels, time to peak, and area under the curve of dogs fed diets formulated with either low or high tannin fava beans without (UF) or with (FM) fermentation, or normal (NP) vs. high (HP) protein commercial diets for 7 days each^*^.

**Item**	**Commercial**			**Low tannin**		**High tannin**			***P*****-value**^**a**^		
	**NP**	**HP**	**SEM**	**UF**	**FM**	**UF**	**FM**	**SEM**	***P***	**FB**	**FM**
Fasting blood glucose level (mmol/L)	3.98	3.99	0.19	3.62	3.87	3.77	3.74	0.16	0.97	0.96	0.37
Peak blood glucose level (mmol/L)	7.02	6.96	0.35	5.69	5.81	6.33	5.80	0.23	0.92	0.15	0.36
Time to peak (min)	37.50	41.25	7.15	31.53	30.09	26.26	32.31	5.56	0.76	0.78	0.68
Area under the curve (mmol/L × min)	321.98	324.38	15.50	273.59	275.07	282.47	265.02	9.26	0.91	0.95	0.41

* *Eight mixed-gender, neutered beagles were fed the NP or HP diets during the first and sixth weeks, respectively. From weeks 2 to 5, using a randomized, crossover, 2 × 2 Latin square design, 4 diets differing in fava bean variety and fermentation were compared as follows: unfermented (UF) high tannin, fermented (FM) high tannin, UF low tannin, and FM low tannin. Values expressed as means (n = 8). SEM = pooled standard error of the mean*.

a *P, P-value between commercial diets with different protein content (NP vs. HP); FB, fava bean (low tannin vs. high tannin); FM, fermentation (UF vs. FM). The interaction between FB and FM was not significant for any of the parameters measured (P > 0.10)*.

### Red and White Blood Cell Count

**Figure 2:**
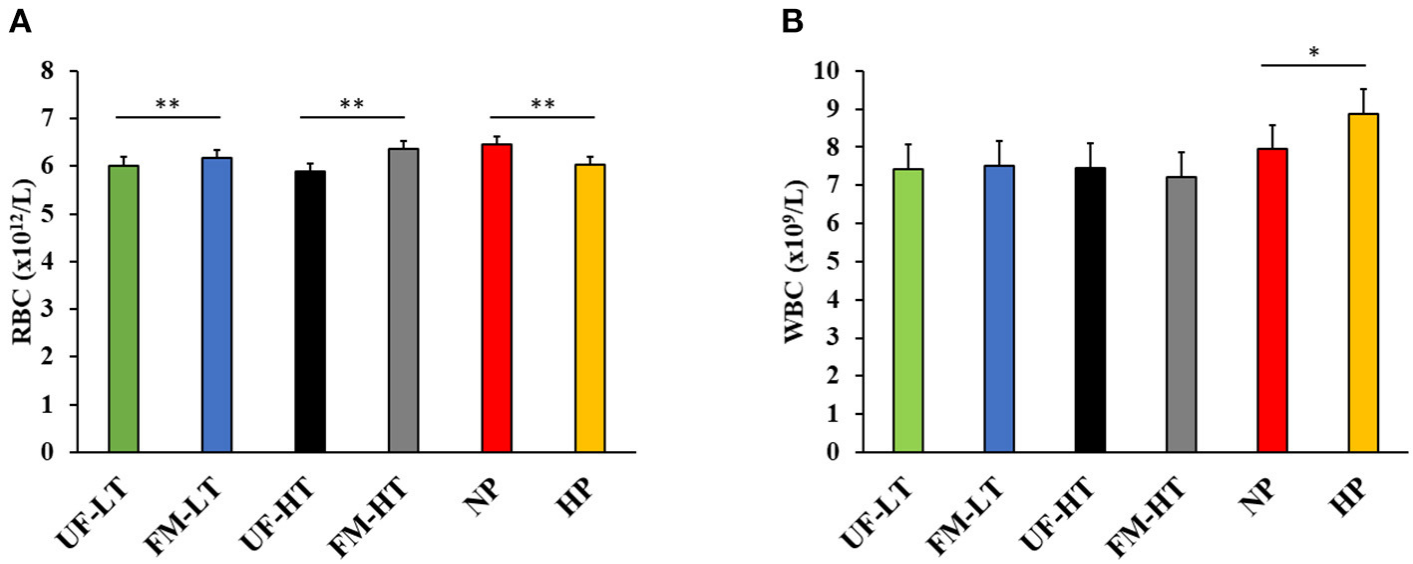
Red [RBC; **(A)**] and white [WBC; **(B)**] blood cell counts in dogs fed either low (LT) or high (HT) tannin fava bean–based diets without (UF) or with (FM) fermentation, or normal vs. high protein commercial diets. Eight mixed-gender, neutered beagles were fed the NP, or HP diets during the first and sixth weeks, respectively. From weeks 2 to 5, using a randomized, crossover, 2 × 2 Latin square design, 4 diets differing in fava bean variety and fermentation were compared as follows: UF-HT, FM-HT, UF-LT, and FM-LT *P < 0.10; **P < 0.05.

White blood cell and RBC of beagles after feeding each diet for 7 days are shown in Figure 2. Dogs fed HP diets showed decreased RBC compared with NP-fed dogs (*P* < 0.05). Conversely, WBC tended to increase in dogs fed HP diets compared with NP-fed dogs (*P* < 0.10). Within fava bean–based diets, FM increased RBC regardless of FB (*P* < 0.05) and no effect (*P* > 0.10) of either FM, FB, or the interaction between FM and FB was observed on WBC.

### Blood Parameters of Hepatic Function

Blood parameters indicative of hepatic function in beagles after feeding test diets for 7 days are shown in Table 3. Within commercial diets, serum cholesterol was increased, while bilirubin parameters (TB, DB, and IB) were decreased in dogs fed HP diets compared with NP-fed dogs (*P* < 0.05). Furthermore, serum ALP and ALT were decreased in dogs fed HP diets compared with NP-fed dogs (*P* < 0.05). Moreover, total serum protein was decreased in dogs fed HP diets compared with NP-fed dogs, primarily due to a decrease in serum globulin, which elevated the albumin:globulin ratio (*P* < 0.05). There was no effect of dietary protein content on GGT, GLDH, CK, and albumin (*P* > 0.10). Among fava bean–based diets, DB was higher in dogs fed LT compared to HT-fed dogs regardless of FM (*P* < 0.05). There was no significant effect (*P* > 0.10) of either FM, FB, or the interaction between FM and FB on any other blood parameter of hepatic function measured.

**Table 3:** Blood parameters of hepatic function in dogs fed diets formulated with either low or high tannin fava beans without (UF) or with (FM) fermentation, or normal (NP) vs. high (HP) protein commercial diets for 7 days each^*^.

**Item**	**Commercial**			**Low tannin**		**High tannin**			***P*****-value**^**a**^		
	**NP**	**HP**	**SEM**	**UF**	**FM**	**UF**	**FM**	**SEM**	***P***	**FB**	**FM**
Cholesterol (mmol/L)	4.17	4.41	0.36	3.00	3.14	3.05	3.01	0.26	0.03	0.61	0.50
TB (μmol/L)	0.76	0.27	0.10	0.67	0.62	0.57	0.53	0.16	<0.01	0.55	0.78
DB (μmol/L)	0.43	0.18	0.07	0.37	0.38	0.28	0.21	0.08	0.01	0.05	0.65
IB (μmol/L)	0.33	0.09	0.07	0.30	0.23	0.20	0.32	0.12	0.02	0.96	0.80
ALP (U/L)	62.87	51.12	10.53	70.62	70.87	74.87	68.25	11.69	0.02	0.80	0.34
GGT (U/L)	0.37	0.68	0.30	2.25	1.62	1.75	2.37	0.74	0.31	0.84	1.00
ALT (U/L)	24.87	20.12	1.23	28.50	29.75	28.37	26.75	3.13	<0.01	0.27	0.89
GLDH (U/L)	4.75	4.50	0.45	5.25	6.12	5.12	4.50	1.22	0.51	0.28	0.87
CK (U/L)	148.25	197.37	19.65	116.27	143.32	165.23	163.52	17.73	0.06	0.10	0.51
Total protein (g/L)	52.75	51.62	1.65	50.75	51.00	51.12	51.37	1.49	0.03	0.80	0.86
Albumin (A; g/L)	31.75	32.75	1.36	32.12	32.25	32.25	32.25	1.32	0.06	0.89	0.89
Globulin (G; g/L)	21.00	18.87	0.76	18.62	18.75	18.75	19.12	0.62	<0.01	0.41	0.62
A:G	1.52	1.75	0.08	1.73	1.74	1.71	1.69	0.08	<0.01	0.52	0.85

* *Eight mixed-gender, neutered beagles were fed the NP or HP diets during the first and sixth weeks, respectively. From weeks 2 to 5, using a randomized, crossover, 2 × 2 Latin square design, 4 diets differing in fava bean variety and fermentation were compared as follows: unfermented (UF) high tannin, fermented (FM) high tannin, UF low tannin, and FM low tannin. Values expressed as means (n = 8). SEM, pooled standard error of the mean; TB, total bilirubin; DB, direct bilirubin; IB, indirect bilirubin; ALP, alkaline phosphatase; GGT, gamma-glutamyl transferase; ALT, alanine aminotransferase; GLDH, glutamate dehydrogenase; CK, creatine kinase*.

a *P, P-value between commercial diets with different protein content (NP vs. HP); FB, fava bean (low tannin vs. high tannin); FM, fermentation (UF vs. FM)*.

### Blood Electrolytes

Blood electrolytes from beagles after feeding each test diet for 7 days are shown in Table 4. Within commercial diets, serum bicarbonate, Ca, and P were increased in dogs fed HP compared with NP-fed dogs (*P* < 0.05). There was no effect (*P* > 0.10) of serum protein content on Na, K, Na:K, Cl, anion gap, or Mg. Among fava bean–based diets, serum P was increased in dogs fed HT compared with LT-fed dogs, regardless of FM (*P* < 0.05). Furthermore, FM tended to increase serum P compared with UF, regardless of FB (*P* < 0.05). There was an interaction between FB and FM for serum K and Na:K (*P* < 0.05). Dogs fed FM-HT diets showed the highest serum K, UF-HT the lowest, with FM-LT and UF-LT being intermediate. Consequently, dogs fed UF-HT diets showed the highest serum Na:K, and FM-HT the lowest, with FM-LT and UF-LT being intermediate. There was no significant effect (*P* > 0.10) of either FM, FB, or the interaction between FM and FB on serum Na, Cl, bicarbonate, anion gap, Ca, and Mg.

**Table 4:** Blood electrolytes (mmol/L) of dogs fed diets formulated with either low or high tannin fava beans without (UF) or with (FM) fermentation, or normal (NP) vs. high (HP) protein commercial diets for 7 days each^*^.

**Item**	**Commercial**			**Low tannin**		**High tannin**			***P*****-value**^**a**^			
	**NP**	**HP**	**SEM**	**UF**	**FM**	**UF**	**FM**	**SEM**	***P***	**FB**	**FM**	**FB × FM**
Na	146.50	147.25	0.54	146.34	145.67	146.62	146.26	0.55	0.24	0.36	0.28	0.71
K	4.80	4.87	0.07	4.65^b, c^	4.62^b, c^	4.56^c^	4.74^b^	0.06	0.52	0.70	0.13	0.01
Na:K	30.50	30.00	0.56	31.26^b, c^	31.49^b, c^	32.19^b^	31.08^c^	0.51	0.54	0.47	0.23	0.04
Cl	113.63	113.88	0.60	112.13	111.54	111.65	112.21	0.65	0.62	0.81	0.97	0.12
HCO_3_-	19.12	20.50	0.38	20.28	19.22	19.92	20.03	0.63	0.04	0.73	0.48	0.38
Anion gap	18.62	17.87	0.47	18.52	19.64	19.53	19.02	0.92	0.26	0.83	0.73	0.35
Ca	2.44	2.49	0.03	2.45	2.46	2.47	2.45	0.03	0.01	0.70	0.37	0.22
*P*	1.49	1.67	0.03	1.34	1.47	1.48	1.53	0.04	<0.01	0.05	0.08	0.42
Mg	0.88	0.81	0.08	0.85	0.82	0.83	0.84	0.01	0.56	0.87	0.55	0.11

* *Values expressed as means (n = 8)*.

a *P, P-value between commercial diets with different protein content (NP vs. HP); FB, fava bean (low tannin vs. high tannin); FM, fermentation (UF vs. FM)*.

b,c *Means within a same row with no common superscript differ significantly (P <0.05)*.

### Blood Urea and Creatinine

**Figure 3:**
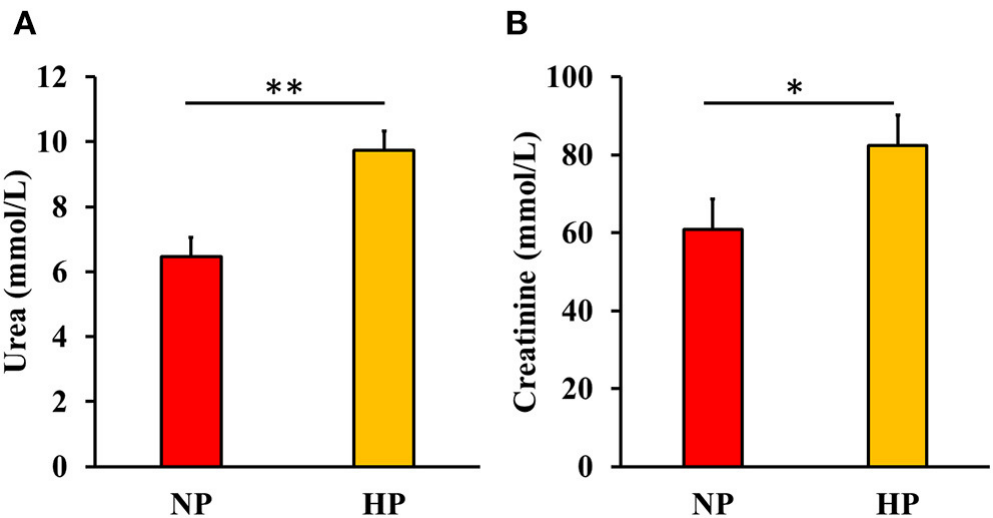
Blood urea **(A)** and creatinine **(B)** content in dogs fed either normal (NP) or high protein (HP) commercial diets. Eight mixed-gender, neutered beagles were fed the NP, or HP diets during the first and sixth weeks, respectively *P < 0.10; **P < 0.05. There was no effect of either fava bean (FB), fermentation (FM), or the interaction between FB and FM on urea and creatinine content (*P* > 0.05).

Blood urea and creatinine of beagles after feeding each test diet for 7 days are shown in Figure 3. Serum urea was increased (*P* < 0.05) while creatinine tended to increase (*P* < 0.10) in dogs fed HP compared with NP-fed dogs. There was no significant effect (*P* > 0.10) of either FM, FB, or the interaction between FM and FB on serum urea and creatinine.

### Digestive Enzymes in Blood

**Figure 4:**
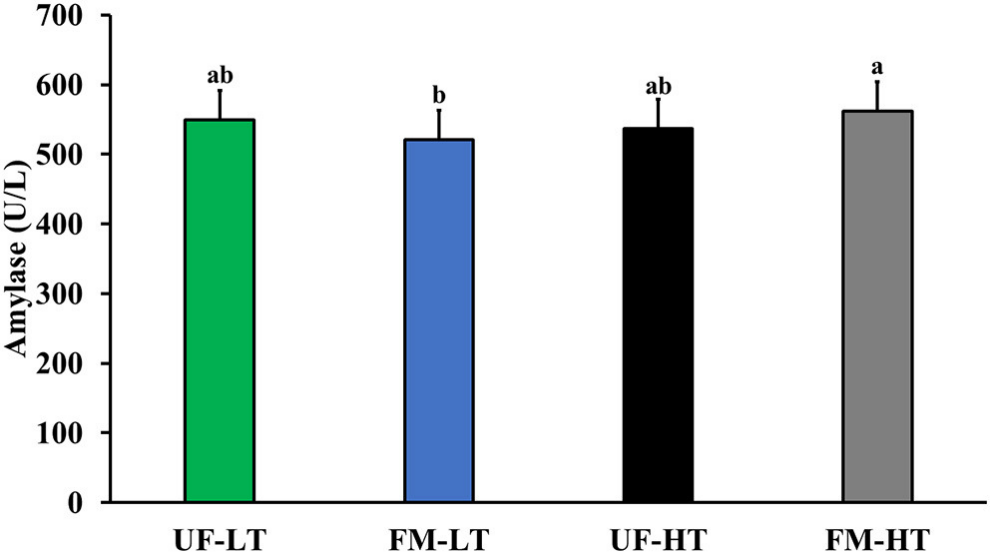
Blood amylase content in dogs fed either low (LT) or high (HT) tannin fava bean–based diets without (UN) or with (FM) fermentation. From weeks 2 to 5, using a randomized, crossover, 2 × 2 Latin square design, 8 mixed-gender, neutered beagles were fed 4 diets differing in fava bean variety and fermentation as follows: UF-HT, FM-HT, UF-LT, and FM-LT. Bars with no common letter (a, b) differ significantly (*P* < 0.05). There was no effect of dietary protein content on serum amylase content (*P* > 0.05).

After 7 days of feeding each test diet to dogs, there was an interaction between FB and FM, where serum amylase was highest in FM-HT, lowest in FM-LT, but intermediate in UF-LT and UF-HT (*P* < 0.05; Figure 4). However, there was no significant effect of either FM or FB on serum amylase (*P* > 0.10). Moreover, dietary protein content did not significantly impact serum amylase (*P* > 0.10).

### Cardiovascular Function

Cardiovascular function parameters of beagles after 7 days of feeding each test diet are shown in Table 5. Dogs fed HP had decreased left ventricular end-systolic volume (ESV) and cardiac output (CO) compared with NP-fed dogs (*P* < 0.05). Dogs fed FM diets tended to have decreased maximum velocity (MV) of passive ventricular filling (the early or E wave) compared with UF-fed dogs regardless of FB (*P* < 0.10). There was no significant effect of protein content or either FM, FB, or the interaction between FM and FB on left ventricular end-diastolic volume (EDV), stroke volume (SV), heart rate (HR), ejection fraction (EF), left ventricular diastolic wall thickness (DWT), left ventricular systolic wall thickness (SWT), systolic blood pressure (SBP), diastolic blood pressure (DBP), flow-mediated dilation (FMD), velocity time integral for ventricular filling (VTI) (E and A wave combined), and MV (A wave) (*P* > 0.10). Moreover, there was no significant effect of dietary treatments on LVID_d_ and LVID_s_ (*P* > 0.10).

**Table 5:** Cardiovascular function parameters of dogs fed diets formulated with either low or high tannin fava beans without (UF) or with (FM) fermentation, or normal (NP) vs. high (HP) protein commercial diets for 7 days each^*^.

**Item**	**Commercial**			**Low tannin**		**High tannin**			***P*****-value**^**a**^		
	**NP**	**HP**	**SEM**	**UF**	**FM**	**UF**	**FM**	**SEM**	***P***	**FB**	**FM**
EDV (ml)	24.25	21.33	2.50	19.36	16.84	16.86	17.33	1.89	0.42	0.60	0.59
ESV (ml)	8.67	4.37	1.32	2.97	2.36	2.59	2.42	0.60	0.05	0.79	0.52
LVID_d_ (cm/BW^1/3^)	0.96	0.88	0.07	0.92	0.85	0.83	0.90	0.08	0.43	0.85	0.99
LVID_s_ (cm/BW^1/3^)	0.47	0.43	0.05	0.37	0.37	0.36	0.36	0.04	0.58	0.79	1.00
SV (ml)	18.62	16.96	1.37	16.39	14.59	14.29	14.91	1.41	0.41	0.53	0.68
CO (L/min)	1.84	1.41	0.14	1.41	1.34	1.41	1.21	0.18	0.04	0.72	0.44
EF (%)	79.38	83.13	3.49	85.88	86.44	85.56	87.31	3.20	0.46	0.89	0.60
HR (bpm)	97.75	93.13	5.05	102.83	91.17	101.29	91.50	5.55	0.53	0.91	0.07
DWT (cm)	0.83	0.79	0.07	0.85	0.89	0.90	0.88	0.05	0.72	0.57	0.90
SWT (cm)	1.52	1.35	0.13	1.50	1.37	1.48	1.39	0.08	0.38	0.99	0.17
SBP (mmHg)	136.12	128.93	5.52	131.31	118.06	127.75	123.25	7.95	0.37	0.91	0.27
DBP (mmHg)	76.88	72.57	6.58	67.42	61.87	63.80	62.50	5.57	0.65	0.79	0.54
FMD (%)	13.25	6.78	4.03	6.88	10.35	6.54	9.66	4.35	0.27	0.90	0.45
VTI (E wave) (cm)	9.87	9.23	2.14	7.49	8.43	9.08	8.97	1.23	0.83	0.40	0.74
VTI (A wave) (cm)	1.10	0.82	0.22	1.02	1.13	1.40	1.21	0.21	0.39	0.30	0.84
MV (E wave) (cm/s)	87.80	82.46	13.30	72.08	65.86	83.00	80.40	6.58	0.76	0.51	0.06
MV (A wave) (cm/s)	44.31	37.03	4.76	40.67	35.75	49.14	41.32	5.21	0.30	0.19	0.23

* *Eight mixed-gender, neutered beagles were fed the NP or HP diets during the first and sixth weeks, respectively. From weeks 2 to 5, using a randomized, crossover, 2 × 2 Latin square design, 4 diets differing in fava bean variety and fermentation were compared as follows: unfermented (UF) high tannin, fermented (FM) high tannin, UF low tannin, and FM low tannin. Values expressed as means (n = 8). SEM, pooled standard error of the mean; EDV, left ventricular end-diastolic volume; ESV, left ventricular end-systolic volume; LVID_d_, left ventricular end diastolic diameter; LVID_s_, left ventricular end systolic diameter; SV, stroke volume; HR, heart rate; CO, cardiac output; EF, ejection fraction; DWT, left ventricle diastolic wall thickness; SWT, left ventricle systolic wall thickness; SBP, systolic blood pressure; DBP, diastolic blood pressure; FMD, flow-mediated dilation; VTI (E wave), velocity time integral (E wave); VTI (A wave), velocity time integral (A wave); MV (E wave), maximum velocity (E wave); MV (A wave), maximum velocity (A wave)*.

a *P, P-value between commercial diets with different protein content (NP vs. HP); FB, fava bean (low tannin vs. high tannin); FM, fermentation (UF vs. FM). The interaction between FB and FM was not significant for any of the parameters measured*.

### Digestibility

Apparent total tract digestibility in beagles fed fava bean–based diets are shown in Table 6. Dogs fed FM diets had decreased fat digestibility and increased non-fiber carbohydrates digestibility compared with UF diets regardless of FB (*P* < 0.05). There was no significant effect of FM, FB, or the interaction between FM and FB on digestibility of crude protein, gross energy, methionine, cysteine, or taurine (*P* > 0.10).

**Table 6:** Apparent total tract digestibility of dogs fed diets formulated with either low or high tannin fava beans without (UF) or with (FM) fermentation for 7 days each^*^.

**Item**	**Low tannin**		**High tannin**			***P*****-value**^**a**^	
	**UF**	**FM**	**UF**	**FM**	**SEM**	**FB**	**FM**
Crude protein	85.74	86.80	85.91	84.74	1.044	0.37	0.95
Fat	91.93	89.15	92.44	89.15	1.262	0.66	0.01
Non-fiber carbohydrates	95.94	96.59	96.09	96.64	0.214	0.76	0.05
Gross energy	92.02	92.19	91.85	91.61	0.347	0.29	0.91
Methionine	85.87	84.56	83.28	83.32	1.299	0.15	0.62
Cysteine	84.38	87.34	88.26	87.34	1.491	0.21	0.50
Taurine	86.68	84.08	84.88	84.87	1.545	0.74	0.41

* *From weeks 2 to 5, using a randomized, crossover, 2 × 2 Latin square design, 8 mixed-gender, neutered beagles were fed 4 diets differing in fava bean variety and fermentation as follows: unfermented (UF) high tannin, fermented (FM) high tannin, UF low tannin, and FM low tannin. Values expressed as means (n = 8). SEM, pooled standard error of the mean*.

a *FB, fava bean (low tannin vs. high tannin); FM, fermentation (UF vs. FM). The interaction between FB and FM was not significant for any of the parameters measured*.

### Plasma Amino Acid Levels

Plasma amino acid concentrations in beagles after 7 days of feeding each test diet are shown in Table 7. Dogs fed HP diets had increased taurine, cystine, and cysteine concentrations compared with NP-fed dogs (*P* < 0.05). There was no significant effect of dietary protein content on plasma methionine concentration (*P* > 0.10). Plasma cysteine was increased in HT-fed dogs compared with LT-fed dogs regardless of fermentation (*P* < 0.05). There was no significant effect of fava bean variety on plasma taurine, cystine, and methionine levels (*P* > 0.05). However, fermentation increased plasma cysteine (*P* < 0.05) and tended to decrease plasma cystine (*P* < 0.10) regardless of fava bean variety. There were no significant interactions between fava bean variety and fermentation on plasma amino acid levels (*P* > 0.10).

**Table 7:** Plasma amino acid levels (nmol/ml) of dogs fed diets formulated with either low or high tannin fava beans without (UF) or with (FM) fermentation, or normal (NP) vs. high (HP) protein commercial diets for 7 days each^*^.

**Item**	**Commercial**			**Low tannin**		**High tannin**			***P*****-value**^**a**^		
	**NP**	**HP**	**SEM**	**UF**	**FM**	**UF**	**FM**	**SEM**	***P***	**FB**	**FM**
Taurine	60.83	116.38	13.576	79.81	74.91	90.88	87.39	9.953	0.01	0.22	0.65
Cystine	10.83	13.33	0.774	16.75	14.75	16.38	13.75	1.467	0.04	0.59	0.08
Cysteine	54.00	472.50	20.401	85.75	137.25	112.62	153.87	35.863	<0.01	0.05	<0.01
Methionine	55.20	51.50	2.203	44.57	45.86	44.67	41.00	2.551	0.26	0.36	0.64

* *Eight mixed-gender, neutered beagles were fed the NP or HP diets during the first and sixth weeks, respectively. From weeks 2 to 5, using a randomized, crossover, 2 × 2 Latin square design, 4 diets differing in fava bean variety and fermentation were compared as follows: unfermented (UF) high tannin, fermented (FM) high tannin, UF low tannin, and FM low tannin. Values expressed as means (n = 8). SEM, pooled standard error of the mean*.

a *P, P-value between commercial diets with different protein content (NP vs. HP); FB, fava bean (low tannin vs. high tannin); FM, fermentation (UF vs. FM). The interaction between FB and FM was not significant for any of the parameters measured*.

## Discussion

The objective of this study was to determine if neutered, mixed-gender, adult beagles fed diets with 30% inclusion of fava bean flour would show altered nutrient digestibility, glucose tolerance, overall health, cardiovascular function, and plasma amino acid levels when contrasted to commercial diets with normal vs. high protein. Fava bean diets had moderate protein levels, but were formulated to be near the AAFCO dietary minimums for methionine (0.33% inclusion) or cystine + methionine (0.65% inclusion) (35). This was done intentionally to cause more rapid changes in sulfur-containing amino acid levels in the dogs and potentially cause early, reversible impairments in cardiac function despite using only a 7-day feeding period for each test diet. All fava bean diets in this study met the cystine + methionine AAFCO minimum, but all were slightly below the minimum for methionine alone due to variations in methionine content of the fava beans from reported literature values that were used for diet formulation (26).

### Lack of Evidence for Toxicity From Fava Beans, Digestibility, Glucose Tolerance, and Antinutritional Factors

Fava beans are pulses, a subset of legumes. Other legumes such as peas have been increasingly included in dog diets as a protein and fiber source (48, 49). Pulse ingredients have been controversially associated with grain-free diets and the occurrence of DCM in dogs (13). Specific to fava beans, however, is the additional association from vicine and convicine antinutritional factors with hemolytic anemia in susceptible humans (50). Worries about potential dog toxicity have prevented its AAFCO approval as a dog food ingredient thus far. Fermentation has been used as a valuable approach to reduce anti-nutritional factors in pulses, including trypsin inhibitors, hemagglutinins, and saponins. Moreover, *Candida* species have the potential to synthesize and increase taurine content (32) as well as improve protein digestibility through its breakdown into amino acids by fermentative microorganisms (51). This current study is the first to show that fermentation with *C. utilis* can successfully reduce the vicine/convicine content in fava bean flour, but had no effect on taurine content, protein digestibility, or amino acid digestibility. Of interest, however, is the observation that fermented fava bean diets both caused increases in plasma cysteine after 7 days of feeding, an effect that does not seem to relate to dietary levels and has no current explanation that should be explored in future studies.

The low-tannin variety used in the present study, Snowdrop, is known to have tannin levels as low as 1% (52), while Florent, which was the high tannin variety used in the present study, has been classified as a normal (higher) tannin genotype in Canada (2). Generally, other antinutritional factors tend to be high in varieties with high tannins, but both fava bean varieties used in the present study had high concentration of the antinutritional factors vicine and convicine. The main issue associated with high tannin content in the diet is related to reduced bioavailability of nutrients in the gastrointestinal tract (53, 54). Despite not evaluating bioavailability in the present study, there was no effect of fava bean variety on digestibility values of any nutrient measured. This implies that despite the potential negative effects of tannins on, specially, protein digestibility (55), we do not have evidence to show a clear detrimental effect of tannins. However, within-animal variation in digestive response to tannins and low sample size may have prevented detection of an impact on digestibility (56). Interestingly, fermentation was able to dramatically reduce both the vicine and convicine content in fava bean–based diets, regardless of variety. In a recent study, fermentation with *L. plantarum* degraded the pyrimidine glycosides in fava bean flour within 48 h, which reduced the toxicity of the fermented fava bean as assessed through *ex vivo* assays on human blood (31). In the present study, there was no impairment in glucose tolerance in dogs fed fermented fava bean (FM) diets as revealed by the lack of effect on glucose baseline and peak levels, time to peak, and AUC. This leads to the conclusion either that fava beans do not have any effect on glucose utilization in dogs or that 7 days is not sufficient to alter glucose utilization. However, it should be noted that the four fava bean test diets in this study were fed sequentially in a crossover design. By the end of these four feeding periods, beagles had been fed fava bean–based diets for a month, with no change in glucose utilization from the previous NP period. Moreover, RBC content was unchanged in dogs fed fava beans compared with the NP diet. Taken together, because anemia is a primary sign of favism and none of the fava bean–based diets caused anemia in the dogs in the current study, it can be concluded that fava beans are not toxic in dogs. While AAFCO requires a 6-month feeding study for fava beans to be approved as a pet food ingredient, this study provides initial indications that they are a safe dog food ingredient.

The present study also provided other indication of fermentation of fava bean flour with *C. utilis* enhancing diet quality and consequently health in dogs. Fermentation significantly decreased the digestibility of fat and increased the digestibility of non-fiber carbohydrates. It has been shown in fish that the concentration of carbohydrates in the gut are inversely related to fat digestibility (57) and this is exacerbated when large amounts of starch are present (58). Surprisingly, amylase content in blood was increased in FM-HT compared with FM-LT with both unfermented diets being intermediate. This indicates that the fermentability of fava bean varieties may be different, which might be associated with the carbohydrate composition, releasing increased or decreased amounts of starch when fermented (59). Finally, dogs fed fermented diets showed increased RBC levels compared with those fed unfermented fava bean diets. Low RBC in young dogs is a common occurrence as RBC lifespan is shorter and young RBC contain less hemoglobin when compared with aging RBC (60). An increased RBC content in fermented diet–fed dogs could be associated with an accelerated production of blood cells and increased health that should be further explored in future studies.

### Effect of High Dietary Protein Diet on Dog Health

The results of the current study using commercial diets also provide indications that high dietary protein can negatively affect overall health of dogs. Previous studies have reported that high protein dog diets may negatively impact gastrointestinal health, pre-disposing dogs to diarrhea (61). While diarrhea was not observed with the HP commercial diet tested in the current study, excessive protein intake has been reported to increase proteinuria and overload kidneys, potentially decreasing the overall health of dogs (62), and this is consistent with the higher serum cholesterol, urea, and creatinine observed with the HP diet in the current study. High serum cholesterol in dogs is consistent with a positive relationship reported between protein intake and cholesterol in humans (63). Moreover, bilirubin measurements (TB, DB, and IB) were decreased in HP-fed dogs compared with NP, which agrees with a recent study showing higher bilirubin concentrations in young pigs fed a protein restricted diet compared with a control diet (64). On the other hand, potential benefits of the HP diet in the current study come from the observed decrease in ALP and ALT in dogs fed HP compared with NP. In dogs, higher ALT content is directly associated with hepatocyte membrane damage and necrosis, whereas ALP is positively associated with biliary stasis (65, 66). Increased ALT and ALP in NP dogs may be interpreted as poor hepatic function (67). It should be noted that while trends for changes in blood parameters can be interpreted as positive or negative, all values for all end-points measured in this study fell within clinical norms and thus all dogs were maintained in a healthy state. Future studies using longer feeding periods are required to determine if trends continue and become clinically significant compared to that observed after 7 days in the current study.

### Cardiac Function, Cysteine, Methionine, and Taurine

One of the main hypotheses of the present study was that pulse-based diets with their higher fiber would cause decreases in plasma taurine, cysteine, or methionine levels, subsequently leading to impaired cardiac contractility or enlargement of the heart consistent with DCM. However, after 7 days of feeding each fava bean–based diet, no significant adverse changes were detected in cardiac or vascular function in the current study. Longer feeding trials are needed to confirm that there are no effects. However, what did change in the short term was ESV that was increased in NP-fed dogs compared with HP-fed dogs, but without changes in ventricular chamber size (LVID). Changes in cardiac chamber size would be surprising after only 7 days, thus a functional change such as impaired contractility and reduce cardiac output (CO) is not expected to explain the higher ESV. In fact, the NP-fed dogs instead had higher CO that may be due to a non-significant trend for both HR and SV to increase. This is consistent with a similar non-significant trend for both systolic and diastolic blood pressure to increase in NP-fed dogs compared with HP-fed dogs, suggesting a generalized increase in sympathetic outflow in the NP-fed dogs. Higher sympathetic tone and blood pressure increases afterload and impairs emptying of the left ventricle at the end of systole (i.e., higher ESV), a reliable indicator of impaired systolic function (68).

The proximate analysis of diets showed no major differences in crude fiber content between laboratory-formulated fava bean–based diets and commercial diets. The main differences among diets were observed for cysteine content, which was low in the fava bean diets. Also, methionine and taurine content were highest in HP diets compared with all fava bean–based diets and the NP diet. If reduced dietary taurine levels were driving cardiac changes, then it would have been expected that the NP diet should have led to adverse cardiac changes and this may agree with what we observed. Future studies should explore longer feeding periods and address whether more susceptible dog breeds than beagles produce a stronger relationship between taurine and cardiac impairment.

The fava bean–based diets and the NP commercial diet tested in the current study all had methionine content that was just below the AAFCO minimum. Dogs can synthesize taurine from cysteine or methionine (69), but all three of these amino acids tend to be low or limiting when plant-based protein sources such as pulses are used. Compounding this problem, pulses are high in resistant starch and fiber. Previous studies in dogs have reported that high fiber diets decrease protein digestibility and increase fecal bile acid excretion in feces (70). Because taurocholate is the major bile salt excreted by dogs, the net effect of high fiber has been reported to deplete taurine and impair digestion of protein that contains cysteine and methionine needed to replace it. However, none of the diets tested caused any significant drop in plasma levels of taurine, cysteine, or methionine and levels remained above the reference range throughout the study (27). In fact, fermented fava beans (both varieties) led to significant increases in plasma cysteine, suggesting a potentially beneficial health effect of fermentation processing. In contrast, the high protein (HP) commercial diet led to higher plasma levels of cysteine and taurine, but no change in plasma methionine compared with NP diet, suggesting that 7 days was sufficient time to cause some alterations of blood levels of these amino acids. Cysteine results, however, should be cautiously interpreted due to its unstable nature and an interfering substance during HPLC analysis of this amino acid that could have led to overestimation of cysteine levels (71). Further analysis using a different method would be necessary to confirm the cysteine results.

## Strength and Limitations

A strength of this study was that the fava bean–based diets that were made in our laboratory were tested against two popular commercial brands, giving a more realistic context to the results. Another strength was the use of a Latin square, crossover design where the same dogs were tested on each diet, reducing variability and increasing power with the small sample size of this experiment. However, an important limitation of this study was the duration, which is insufficient to cause major structural cardiac changes, but was long enough to change plasma levels of sulfur-containing amino acid levels, at least in response to high dietary protein. Moreover, the results of this study using young, healthy adult beagles may not apply to older, large breed dogs with genetic susceptibility to taurine deficiency. However, we would predict that changes in taurine and sulfur-containing amino acids would be even more pronounced in these breeds or in older dogs. A last limitation of the present study was the experimental design which does not allow the statistical comparison between commercial and fava bean-based diets.

## Conclusion and Implications

Most importantly, fava bean–based diets did not cause hemolytic anemia and did not alter glucose handling in dogs after 7 days of feeding, thus fava beans appear safe as a dog food ingredient. In contrast, the high-protein grain-free commercial diet adversely altered blood chemistry compared with the normal protein, grain-containing commercial diet we tested. Moreover, the normal protein, grain-based diet appeared to cause excess sympathetic tone, a trend that if it were to continue with long-term feeding, might lead to adverse changes in cardiac health that are distinct from DCM. On the other hand, fermentation with *C. utilis* looks promising to reduce antinutritional factors and potentially improve health through improvements in nutrient digestibility and increased RBC levels in dogs. Studies using longer feeding periods are needed to determine whether these short-term changes are sustained to produce clinically significant changes in dogs.

## Data Availability Statement

The raw data supporting the conclusions of this article will be made available by the authors, without undue reservation.

## Ethics Statement

The animal study was reviewed and approved by University of Saskatchewan Animal Research Ethics Board.

## Author Contributions

LGR, ML, and LW designed the study. LGR, TM, and CQ conducted the study. LGR and LAR performed data analysis. LGR and LW wrote the article. LW was responsible for final content of the article. All authors contributed to the interpretation of the results throughout the study and have read and approved the article.

## Conflict of Interest

LAR was affiliated with Prairie Swine Centre (Saskatoon, SK, Canada) where he used the facility to do his graduate research. The authors also declare that this study received funding or in-kind support from the Saskatchewan Pulse Growers, Western Grains Research Foundation, Alliance Grain Traders (Saskatoon, SK, Canada) and Horizon Pet Foods (Rosthern, SK, Canada). The funders were not involved in the study design, collection, analysis, interpretation of data, the writing of this article, or the decision to submit it for publication.

## Abbreviations

ALP: alkaline phosphatase
ALT: alanine aminotransferase
AUC: area under the curve
CK: creatine kinase
CO: cardiac output
CP: crude protein
DCM: dilated cardiomyopathy
DB: direct bilirubin
DBP: diastolic blood pressure
DWT: left ventricular diastolic wall thickness
EDV: left ventricular end-diastolic volume
EF: ejection fraction
ESV: left ventricular end-systolic volume
FM: fermented
FMD: flow-mediated dilation
G6PD: glucose-6-phosphate dehydrogenase
GGP: gamma-glutamyltransferase
GLDH: glutamate dehydrogenase
HP: high protein
HR: heart rate
HT: high-tannin fava bean variety
IB: indirect bilirubin
LT: low-tannin fava bean variety
LVID_d_: left ventricular end-diastolic diameter
LVID_s_: left ventricular end-systolic diameter
MV: maximum velocity
NP: normal protein
RBC: red blood cell
SBP: systolic blood pressure
SV: stroke volume
SWT: left ventricular systolic wall thickness
TB: total bilirubin
UF: unfermented
VTI: velocity time integral
WBC: white blood cell.
