# Supplementary material for: The Effect of Fermentation of High- or Low-Tannin Fava Bean on Glucose Tolerance, Body Weight, Cardiovascular Function, and Blood Parameters in Dogs After 7 Days of Feeding: Comparison With Commercial Diets With Normal vs. High Protein
Source: Front Vet Sci. 2021 May 11;8:653771. doi: 10.3389/fvets.2021.653771 (PMC8144709; doi:10.3389/fvets.2021.653771)
Supplement: Supplementary file 1 [file Data_Sheet_1.docx]

Supplementary Material

**Supplementary Table S1.** Diet formulation for lab-formulated fava bean diets (%).

| **Ingredient** | **Unfermented** | | **Fermented** | | |  |
| --- | --- | --- | --- | --- | --- | --- |
|  | **Low tannin** | **High tannin** | | **Low tannin** | **High tannin** | |
| Wheat flour | 53.28 | 53.20 | | 53.28 | 53.20 | |
| Low tannin fava bean fermented | - | - | | 30.00 | - | |
| Low tannin fava bean unfermented | 30.00 | - | | - | - | |
| High tannin fava bean fermented | - | - | | - | 30.00 | |
| High tannin fava bean unfermented | - | 30.00 | | - | - | |
| Turkey meal | 11.74 | 13.04 | | 11.74 | 13.04 | |
| Canola oil | 1.40 | 1.00 | | 1.40 | 1.00 | |
| Celite^TM^ | 1.00 | 1.00 | | 1.00 | 1.00 | |
| Vitamin mixture | 1.00 | 1.00 | | 1.00 | 1.00 | |
| Mineral mixture | 0.10 | 0.10 | | 0.10 | 0.10 | |
| Salt | 0.30 | 0.30 | | 0.30 | 0.30 | |
| Dicalcium phosphate | 0.53 | 0.26 | | 0.53 | 0.26 | |
| Calcium carbonate | 0.55 | - | | 0.55 | - | |
| Choline chloride | 0.10 | 0.10 | | 0.10 | 0.10 | |

**Supplementary Table S2.** Ingredient composition of normal and high protein commercial diets. Ingredients are listed in order of decreasing inclusion.

| **Normal protein commercial diet** |
| --- |
| Whole grain corn, meat and bone meal, corn gluten meal, beef fat naturally preserved with mixed-tocopherols, soybean meal, poultry by-product meal, chicken, egg and chicken flavor, whole grain wheat, animal digest, salt, calcium carbonate, potassium chloride, mono and dicalcium phosphate, L-Lysine monohydrochloride, choline chloride, zinc sulfate, ferrous sulfate, manganese sulfate, copper sulfate, calcium iodate, sodium selenite, Vitamin E supplement, niacin (Vitamin B-3), Vitamin A supplement, calcium pantothenate (Vitamin B-5), pyridoxine hydrochloride (Vitamin B-6), Vitamin B-12 supplement, thiamine mononitrate (Vitamin B-1), Vitamin D-3 supplement, riboflavin supplement (Vitamin B-2), menadione sodium bisulfite complex (Vitamin K), folic acid (Vitamin B-9), biotin (Vitamin B-7), Yellow 6, Yellow 5, Red 40, Blue 2, garlic oil. |
| **High protein commercial diet** |
| Chicken Meal, Turkey Meal, Salmon Meal, De-Boned Chicken, De-Boned Turkey, De-Boned Trout, Potatoes, Chicken Fat (Preserved With Mixed Tocopherols), Peas, Tapioca, Lentils, Duck Meal, Chickpeas, Natural Chicken Flavor, Whole Dried Egg, Apples, Herring Meal, Flaxseed, Salmon Oil, Alfalfa, De-Boned Duck, De-Boned Salmon, Sweet Potatoes, Potassium Chloride, Pumpkin, Carrots, Bananas, Blueberries, Cranberries, Broccoli, Blackberries, Squash, Papayas, Pomegranate, Dried Chicory Root, Dried Lactobacillus Acidophilus Fermentation Product, Dried Enterococcus Faecium Fermentation Product, Dried Aspergillus Oryzae Fermentation Extract, Dried Bacillus Subtilis Fermentation Extract, Choline Chloride, Vitamins (Vitamin A Supplement, Vitamin D3 Supplement, Vitamin E Supplement, Niacin, L-Ascorbyl-2-Polyphosphate (A Source Of Vitamin C), D-Calcium Pantothenate, Thiamine Mononitrate, Beta-Carotene, Riboflavin, Pyridoxine Hydrochloride, Folic Acid, Biotin, Vitamin B12 Supplement), Minerals (Zinc Proteinate, Iron Proteinate, Copper Proteinate, Zinc Oxide, Manganese Proteinate, Copper Sulphate, Ferrous Sulphate, Calcium Iodate, Manganous Oxide, Selenium Yeast), Sodium Chloride, Taurine, Yucca Schidigera Extract, Dried Rosemary, Green Tea Extract, Peppermint, Parsley, Rosehips, Zedoary, Dandelion, Chamomile, Ginger, Fennel, Turmeric, Juniper Berries, Licorice, Marigold Extract, Cardamom, Cloves. |

**Supplementary Table S3.** Proximate composition (%, dry matter basis) of lab-formulated fava bean diets compared to commercial diets.

| **Item** | **Unfermented** | | **Fermented** | | **Commercial** | |
| --- | --- | --- | --- | --- | --- | --- |
|  | **Low tannin** | **High tannin** | **Low tannin** | **High tannin** | **Normal protein** | **High protein** |
| Moisture | 9.53 | 9.11 | 7.80 | 9.10 | 7.70 | 5.84 |
| Dry matter | 90.47 | 90.89 | 92.20 | 90.90 | 92.30 | 94.16 |
| Crude protein | 27.42 | 27.25 | 27.94 | 27.98 | 24.64 | 40.73 |
| Crude fiber | 1.05 | 0.47 | 0.50 | 0.52 | 0.79 | 1.05 |
| Fat | 2.71 | 2.51 | 2.07 | 2.15 | 13.10 | 17.38 |
| Ash | 7.20 | 6.74 | 7.01 | 6.67 | 7.56 | 10.07 |
| Cystine | 0.35 | 0.63 | 0.48 | 0.57 | 0.62 | 0.15 |
| Methionine | 0.32 | 0.29 | 0.31 | 0.30 | 0.36 | 0.61 |
| Taurine | 0.06 | 0.06 | 0.06 | 0.06 | 0.04 | 0.23 |
| Non-fiber carbohydrates | 60.72 | 62.12 | 61.56 | 61.77 | 52.99 | 29.83 |
| Total digestible nutrients | 82.85 | 83.88 | 83.57 | 83.90 | 82.73 | 79.94 |
| Metabolizable energy (kcal/g) | 3.72 | 3.80 | 3.76 | 3.80 | 4.04 | 4.09 |
| Vicine (mg/g) | 1.76 | 1.77 | 0.36 | 0.61 | - | - |
| Convicine (mg/g) | 0.50 | 0.58 | 0.12 | 0.22 | - | - |
